# A systematic review of procedural modalities in the treatment of notalgia paresthetica

**DOI:** 10.1111/srt.13723

**Published:** 2024-05-02

**Authors:** Mohammad Ali Nilforoushzadeh, Yekta Ghane, Nazila Heidari, Hanieh Azizi, Fatemeh Fathabadi, Niloufar Najar Nobari, Amirhossein Heidari

**Affiliations:** ^1^ Skin Repair Research Center Shahid Beheshti University of Medical Sciences Tehran Iran; ^2^ Skin and Stem Cell Research Center Tehran University of Medical Sciences Tehran Iran; ^3^ School of Medicine Tehran University of Medical Sciences Tehran Iran; ^4^ School of Medicine Iran University of Medical Sciences Tehran Iran; ^5^ Faculty of Medicine Tehran Medical Sciences Islamic Azad University Tehran Iran

**Keywords:** notalgia paresthetica, procedural, pruritus, systematic review, treatment

## Abstract

**Background:**

Notalgia paresthetica (NP) is a rare condition characterized by localized pain and pruritus of the upper back, associated with a distinct area of hyperpigmentation. Given the lack of standardized treatment and the uncertain efficacy of available options, applying procedural methods is of growing interest in treating NP.

**Aims:**

We sought to comprehensively evaluate the role of procedural treatments for NP.

**Methods:**

We systematically searched PubMed/Medline, Ovid Embase, and Web of Science until November 14th, 2023. We also performed a citation search to detect all relevant studies. Original clinical studies published in the English language were included.

**Results:**

Out of 243 articles, sixteen studies have reported various procedural modalities, with or without pharmacological components, in treating NP. Pharmacological procedures, including injections of botulinum toxin, lidocaine, and corticosteroids, led to a level of improvement in case reports and case series. However, botulinum toxin did not show acceptable results in a clinical trial. Moreover, non‐pharmacological procedures were as follows: physical therapy, exercise therapy, kinesiotherapy, acupuncture and dry needling, electrical muscle stimulation, surgical decompression, and phototherapy. These treatments result in significant symptom control in refractory cases. Physical therapy can be considered a first‐line choice or an alternative in refractory cases.

**Conclusion:**

Procedural modalities are critical in the multidisciplinary approach to NP, especially for patients who are refractory to topical and oral treatments. Procedural modalities include a spectrum of options that can be applied based on the disease's symptoms and severity.

**Abbreviations**
BTX: botulinum toxin
DN4: douleur Neuropathique 4
EMS: electrical muscle stimulation
NIH: National Institute of Health
NP: notalgia paresthetica
Rbtx‐B: rimabotulinum toxin type B
SNAP‐25: synaptosomal‐associated protein, 25 kDa
TENS: transcutaneous electrical nerve stimulation
UVB: ultraviolet B radiation
VAS: visual analog scale

## 1 INTRODUCTION

Notalgia paresthetica (NP) is a type of neuropathy in the peripheral nervous system commonly observed in older females.^1^ NP is identified by chronic pruritus of the medial scapular borders, often accompanied by pain and sensory abnormalities, such as stinging, numbness, or tingling.^2^ The affected region might be linked to a darkened patch, typically resulting from persistent scratching and rubbing to alleviate discomfort. Although the exact etiology of NP is still unknown, the condition may result from compression or damage to dorsal spinal nerves, particularly the upper segments spanning from T2 to T6.^3^ In addition, various peripheral causes contribute to NP and can be categorized as physical, anatomical, genetic, metabolic, or infectious. Patients with NP do not develop primary lesions; however, secondary changes are seen as a result of continuous scratching. Moreover, the distinctive histopathological features of NP illustrated post‐inflammatory melanosis, hyperkeratosis, and an inflammatory melanophage dermal infiltrate.

NP diagnosis is based on the patient's history and physical examination.^4^ Consequently, a misdiagnosis of NP adversely impacts the patient's quality of life, including their emotional well‐being, sleep quality, and autonomy in managing their condition. Hence, having an accurate diagnosis and receiving effective treatment is imperative. The primary treatment for NP involves oral agents. Medications targeting gamma‐amino‐butyric acid (GABA), serotonin, histamine, and inflammation have been explored. Among these treatments, gabapentin is the most effective option in alleviating itching.^5^ Additionally, topical medications are utilized for NP treatment, such as capsaicin, steroids, anesthetics, amitriptyline/ketamine, and doxepin. Capsaicin stands out as the most frequent topical medication for NP with different administration routes, including cream, gel, lotion, solution, or patch with diverse concentrations. Topical capsaicin can provide immediate relief, but its effectiveness varies significantly over time.^6^ Topical steroids exhibit varying effectiveness and prove beneficial only when inflammation is present. Anesthetics, amitriptyline/ketamine, and doxepin are also not commonly employed in treating NP.^7^

In addition to pharmacological medications, the utilization of procedural therapeutic options for NP treatment has increased in recent years. Initial reports indicated that botulinum toxin (BTX) could be a viable option, significantly improving itching.^8^ Moreover, local and intralesional injection of different substances, narrow‐band ultraviolet B radiation (UVB), excimer lamp, and needling are recognized as alternative procedures that could be promising in treating NP.^2^, ^7^, ^9^, ^10^, ^11^ However, these approaches are currently unapproved for NP due to insufficient evidence regarding their effectiveness and safety. Consequently, assessing the effectiveness and safety profile of the available procedures can help clinicians find the best approach for treating NP patients. The current systematic review aims to evaluate the efficacy and safety of procedural treatment options for patients with NP.

## 2 MATERIAL AND METHODS

The current systematic review is based on the Preferred Reporting Item for Systematic Reviews and Meta‐Analysis (PRISMA) 2020 statement.^12^ The checklists can be found in the Supplementary documents (Tables S1 and S2).

### 2.1 Search strategy

A comprehensive systematic search was conducted across three databases, PubMed/Medline, Ovid Embase, and Web of Science, from their inception until November 14th, 2023. A full list of keywords and MeSH terms is available in the Supplementary documents (Table S3).

### 2.2 Eligibility criteria and study selection

Clinical studies available in English full text were included in this systematic review. The source populations eligible for this study were individuals with NP of any age and gender who underwent any procedural treatment. Studies that used non‐procedural treatments, review articles, guidelines, book chapters, in vitro/ex vivo studies, and animal studies were not eligible for inclusion.

### 2.3 Data extraction

Three investigators independently conducted the data extraction process for the selected articles (YG, HA, and FF). The process was as follows: (I) Extraction of study and patient characteristics, including author, year, design, sample size, gender, previous treatments, and type of procedural treatment. (II) Extraction of results, including outcome measurements, efficacy, safety, adverse events, and follow‐ups. Moreover, any disagreements were resolved by the corresponding authors during the data extraction process.

### 2.4 Risk of bias assessment

Three reviewers (YG, NH, and AH) independently appraised the included studies' bias risk and methodological quality of included studies. For these evaluations, we used the National Institute of Health (NIH) Quality Assessment Tool for Clinical Trials,^13^ NIH for Before‐After (Pre‐Post) Studies with No Control Group,^14^ NIH for Observational Cohort and Cross‐Sectional Studies,^15^ and Murad et al.^16^ methodological quality assessment for the case series and case reports. The risk of bias assessment results are illustrated in Tables S4–S7.

## 3 RESULTS

### 3.1 Search results

**FIGURE 1:**
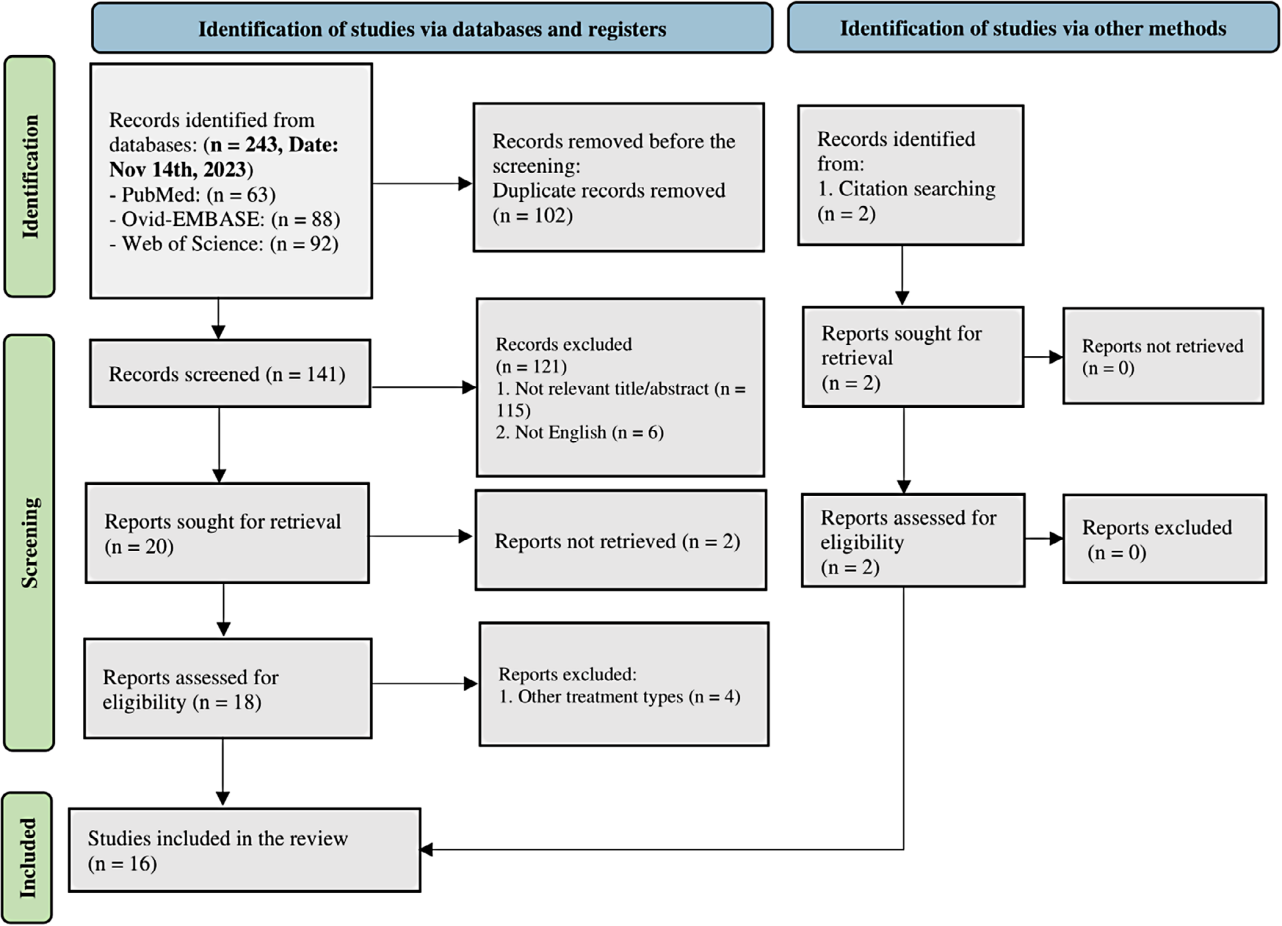
PRISMA 2020 flow diagram for new systematic reviews, which included searches of databases, registers, and other sources.

A total of 243 records were identified in a search conducted until November 14th, 2023. A number of 102 duplicates were found and removed subsequently. In the first and second steps of the screening process, the titles and abstracts of the remaining 141 records were reviewed. Disagreements that arose during the screening process were resolved through consensus with the third reviewer or the corresponding authors. The full texts of 14 articles were reviewed according to predefined eligibility criteria in the final phase of the screening. Moreover, two additional studies were included after the citation search. Ultimately, 16 articles were included for data extraction in this systematic review. The PRISMA flowchart of the inclusion process is depicted in Figure 1.

### 3.2 Characteristics of eligible studies

A total of 16 studies were included in this systematic review, including one randomized controlled trial study, one pilot study, one prospective and two retrospective cohort studies, one cross‐sectional study, seven case series, and three case reports. A total of 198 patients were included; 120 of them received pharmacological procedures, and 78 received non‐pharmacological procedures. Regarding pharmacological procedures, 67.5% (*n* = 81) were treated with lidocaine, 25.8% (*n* = 31) with BTX‐A, 2.5% (*n* = 3) with rimabotulinum toxin type B (Rbtx‐B), and 4.1% (*n* = 5) with triamcinolone acetonide injections. In studies assessing non‐pharmacological procedures, 33.33% (*n* = 26) were treated with physical therapy, 20.51% (*n* = 16) with acupuncture, 14.1% (*n* = 11) with excimer lamp, 10.25% (*n* = 8) with dry needling, 6.41% (*n* = 5) with narrow‐band UVB, 5.12% (*n* = 4) with electrical muscle stimulation (EMS), 5.12% (*n* = 4) with exercise therapy, 3.84% (*n* = 3) with kinesiotherapy, and 1.28% (*n* = 1) with surgical decompression (Table 1).

**TABLE 1:** Characteristics of eligible studies utilized procedural modalities in the treatment of notalgia paresthetica.

Study ID	Design of study	Sample size	Gender	Previous treatment (s)	Treatment	Outcome measurement	Efficacy	Safety and adverse events	Follow‐up
**Karasel, 2022**	Retrospective cohort study	25	F	NA	44% (*n* = 11) of the patients undertook physical therapy (15 sessions over 3 weeks), including hot pack (20 min), TENS (conventional 70−100 Hz, 20 min), and ultrasound therapy (1w/cm^2^, min), 28% (*n* = 7) of the patients with dry needle therapy (5 times over 3 weeks), 16% (*n* = 4) of the patients undertook a home exercise therapy program(stretching and resistance exercises), 12% (*n* = 3) of the patients with kinesiotherapy (5 times over 3 weeks)	VAS score DN4 score	Significantly decreased in VAS and DN4 score from 7 to 2 decreased pain levels in patients by TENS therapy, significant success with kinesiology taping in 9 patients, reduction of average VAS scores for both itching and pain	No AEs	NA
**Fonda‐Pascual, 2021**	Prospective cohort	11	F: 81.8%	Topical corticosteroids (*n* = 8), topical capsaicin (*n* = 4), topical anesthetics (*n* = 2), antihistamines (*n* = 4), botulinum toxin(*n* = 1)	Excimer lamp 308‐nm with a median number of sessions of 4.27 The mean accumulative dose of 1281.81 mJ/cm^2^	VAS score	A global reduction in VAS and pruritus (from mean 8.18 to 3.09) The median number of sessions to reach pruritus remission is 4.11 Pruritus remission in 9 patients	No AEs	No relapse until six months after therapy
**Özcan, 2020**	Case series	5	F	Topical corticosteroid (*n* = 2)	Intralesional triamcinolone acetonide injections (10 mg/mL; 0.1 mL/cm^2^) every 3 weeks for a maximum of four treatments in three patients and a total of two injections in two patients	VAS score	100% improvement in itch scores in 33% of patients Resolution of numbness and tingling in two patients after the first injection	Self‐limited skin atrophy in all patients after the second injection	The severity of the itch returned to the original level 8 weeks after the last injection in 1 patient Partial resolution, as well as reduction in the size of hyperpigmentation
**Mülkoǧlu, 2020**	Cross‐sectional	NP group: 45 Control group (dorsalgia without NP): 35	NP group: F: 87% Control group: F: 60%	NA	Local lidocaine injections	VAS score	DecreasedVAS‐pruritus score at the 2nd and 4th‐week of follow‐up	No AEs	Relief of pain and pruritus at three months of follow‐up
**Bağcıer, 2020**	Case report	1	M	Topical corticosteroid and antihistamine	Dry needling 3 Sessions	DN4 score 5‐D itch score	Decrease of DN4 and 5‐D itch scores	No AEs	NA
**Chtompel, 2017**	Case report	1	F	Antihistamines and topical hydrocortisone cream, pregabalin, methadone, orphenadrine, cyclobenzaprine, methocarbamol, baclofen, nabilone, acetaminophen, mirtazapine	Lidocaine IV infusion1 mg/kg bolus followed by 4 mg/kg over one hour for three injections	Clinical signs and symptoms	90% improvement in itch, which lasted seven days after the first infusion and ten days after the second infusion 20% reduction in the itch severity after recurrence compare to the baseline Reduction in lower limb pain with an NRS of 1/10 for 14 days after the third injection	No AEs	4‐week intervals
**Pérez‐Pérez, 2014**	Case series	5	F: 80%	Pt1: topical corticosteroids, oral antihistamines Pt2: topical corticosteroids, pregabalin, oxcarbazepine, TENS Pt3: topical corticosteroids, capsaicin, NB—UV‐B, oxcarbazepine, physical therapy Pt4: topical corticosteroids Pt5: topical corticosteroids, oral antihistamines	Intradermal botulinum toxin type A	Clinical signs and symptoms	Failure in disappearing of pruritus in all patients, relapse of disease in 3 patients after 1‐month injection, worsening pruritus in 2 patients	No AEs	NA
**Maari, 2014**	Double‐blind randomized controlled trial	20	F	NA	G1 (*n* = 10): botulinum toxin A injections of 0.1 mL (50 U/mL) for the hyperpigmented area, the maximum intradermal dose of 200 U BTX‐A for non‐hyperpigmentation area based on patients delimited pruritis G2 (*n* = 10): saline placebo	VAS score	No statistically significant mean difference in pruritus VAS between patients treated with BTX‐A (0.72 ± 2.97) and those given a placebo (0.91 ± 3.8) 8 weeks after the treatment	No AEs	NA
**Grogan, 2011**	Case series	3 (a total of 18 patients, 3 with NP)	NA	NA	Intradermal injections of rimabotulinum toxin B dosages ranged from 1000−4000 units total per treatment session (average: 2250 units)	The subjective account of pain and VAS score	Substantial reductions in pain and paresthesias in most patients, reduction in VAS scores by an average of 4.3 points before/after injections	No AEs	NA
**Williams, 2010**	Case report	1	F	Transcutaneous electrical nerve stimulation units, postural training, yoga, bracing, anti‐inflammatory, opiates	Surgical decompression	Clinical signs and symptoms	Original pain was reduced by 50% in the 1st week, discomfort in the paraspinal muscles and the initial pain alleviated in the 2nd week, free of symptoms four months after surgery	No AEs	NA
**Wallengren, 2010**	Case series	4 (a total of 6 patients, 4 with NP)	F	Pt1: topical capsaicin 0.025% and cutaneous field stimulation Pt2: topical capsaicin, acupuncture Pt3: topical capsaicin Pt4: topical capsaicin, acupuncture	Intracutaneously injections of botulinum toxin type A Pt1: injection dose of 100 U Pt2: injection dose of 30 U Pt3: injection dose of 18 U Pt4: injection dose of 40 U	VAS score	A mean improvement of 28% in VAS score	No AEs	18 months of follow up Pt1: VAS of 45%
**Pérez‐Pérez, 2010**	Case series	5	F: 80%	Pt1: pramocaine capsaicin Pt2: none Pt3: topical pramocaine, corticoids Pt4: topical capsaicin Pt5: topical capsaicin; oral oxcarbazepine, gabapentin	Narrow‐band UVB Pt1: cumulative dose of 4.41 Pt2: cumulative dose of 39.89 Pt3: cumulative dose of 55.17 Pt4: cumulative dose of 34.19 Pt5: cumulative dose of 35.18	Clinical signs and symptoms	Pt1: pruritus changed from 8 to 0 Pt2: pruritus changed from 10 to 5 Pt3: pruritus changed from 5.6 to 2 Pt4: pruritus changed from 7 to 0 Pt5: pruritus changed from 6 to 3.Pt4: pruritus changed from 7 to 0	No AEs	NA
**Wang, 2009**	Case series	4	M: 75%	Pt1: 60 and 120 mg of duloxetine Pt2: amytriptyline, valium, Skelaxin, fentanyl,dilaudid, methadone, gabapentin, tizanidine Pt3: topiramate and over‐the‐counter analgesics Pt4: gabapentin, desipramine, multiple opioids	EMS at 70 Hz with a pulse width of 300µs be 30 seconds on and 30 seconds off for 15 minutes twice a day	Clinical signs and symptoms	Relief of the pain shortly after beginning EMS and recurrence of pain with stopped stimulation for an extended period in all patients Pt1: significant relief of pain but recurrence of the pain after stopping EMS Pt2: pain relief as eight on a scale of 10 Pt3: 70% relief of symptoms within the first 2 weeks of use to the serratus anterior muscle Pt4: steady worsens the pain after discontinuing stimulation	No AEs	Pt1: significant improvement in the quality of life after 20 months of follow‐up Pt2: consistently use the stimulator after nine months of follow‐up Pt3: continues to use the stimulator intermittently after nine months of follow‐up Pt4: using the stimulator intermittently, and positive effects persist after 20 months follow‐up
**Weinfeld, 2007**	Case series	2	F	Pt1: moisturizers and topical corticosteroids Pt2: topical class I steroids, pramoxine hydrochloride cream, 1%, and doxepin hydrochloride cream, 1%	Intradermal of botulinum toxin type A Pt1: 16 U dose of botulinum toxin Pt2: 24 U dose of botulinum toxin	Clinical signs and symptoms	Pt1: near complete resolution of pruritis and hyperpigmentation on the backPt2: Significant improvement after the first injection	No AEs	18 months of follow up Pt1: symptom‐free until the last follow‐up Pt2: no scratching despite some intermittent pruritus; starting a second treatment of 48U resulted in the disappearance of symptoms as well as a decrease in the size and color of hyperpigmentation
**Savk, 2007**	Pilot trial	15	F: 73.3%	NA	Transcutaneous electrical nerve stimulation TENS high‐frequency (50−100 Hz), five sessions a week for 2 weeks totaling ten sessions, 20 min duration with a pulse width of 40−75 µs	Scale of 0 to 10	Pruritus score at the end of the first week was 7.67 ± 2.02 (range, 5−10); by the end of the second week the mean pruritus score was 6.80 ± 2.73(range, 4−11)	A slight worsening of pruritus in 1 patient	NA
**Stellon, 2002**	Retrospective cohort study	16	NA	Variety of steroid or antihistamine‐based creams or oral antihistamines (*n* = 7)	Acupuncture with 1 to 3‐inch 32G needles every 1 to 2 weeks	VAS score	Complete relief from itching in 12 patients, partial relief in four patients	NA	Relapse in six patients over a follow‐up of 7 months

**Abbreviations**: AEs, adverse effects; DN4, douleur neuropathique 4; EMS, electrical muscle stimulation; F, female; IV, intravenous; M, Male; NA, not attributable; NRS, numerical rating scale; Pt, patient; TENS, transcutaneous electrical nerve stimulation; U, unit; UVB, ultraviolet B radiation; VAS, visual analog scale.

### 3.3 Pharmacological procedures

A total of eight articles have investigated the efficacy and safety of pharmacological procedures, including four articles on BTX‐A injection, one study on Rbtx‐B injection, two articles on lidocaine injection, and one study on intralesional triamcinolone acetonide injection.

In a study conducted by Pérez‐Péreze et al.^2^ on five patients with NP, none of the patients experienced a decrease in pruritus; the disease relapsed in three patients after one month, and pruritus was exacerbated in two patients. According to a randomized controlled trial carried out by Maari et al.^17^ on 20 subjects suffering from NP, there was no statistically significant difference in pruritus Visual analog scale (VAS) between patients treated with BTX‐A and those given a placebo after eight weeks of treatment. In another study performed by Wallengren et al.,^8^ four patients underwent various units of BTX‐A. The findings revealed a mean improvement of 28% in the VAS score. Of two patients who reported improvement after six weeks, one reported a VAS of 45%, while the other was still free from itch after 18 months of follow‐up. Weinfeld et al.^18^ investigate two patients receiving different BTX‐A units. One of them remains completely symptom‐free after more than 18 months following treatment, and the hyperpigmentation on her back was barely perceptible. Moreover, the other case showed significant improvement in the clinical manifestations after the first injection. Further, an investigation by Grogan et al.^19^ on three cases with NP who were taking intradermal Rbtx‐B depicted a substantial reduction in pain and paresthesia in most patients as well as a reduction in VAS scores by an average of 4.3 points before and after injections. In terms of safety, all types of BTX demonstrated a favorable safety profile without causing adverse events.

Additionally, two studies evaluated the lidocaine efficacy and safety in NP cases. Regarding the study by Mülkoğlu et al.,^7^ the VAS‐pruritus score decreases in the 2nd and 4th weeks following lidocaine injection. Moreover, Chtompel et al.^20^ reported a case with NP who demonstrated a 90% improvement in itch, lasting seven days after the first infusion and ten days after the second infusion, following lidocaine IV injection. The patient also experienced a 20% reduction in itch severity after recurrence compared to the baseline.

Intralesional triamcinolone acetonide was found to be effective in a case series reported by Özcan et al.,^11^ with respect to 100% improvement in itch scores in 33% of patients. Also, numbness and tingling in two patients after the first injection were reduced. Regarding safety, self‐limited skin atrophy occurs in all patients as an adverse event.

### 3.4 Non‐pharmacological procedures

A total of eight studies have evaluated non‐pharmacological procedures’ efficacy and safety in the management of NP. In a study conducted by Karasel et al.,^21^ 11 patients underwent different types of physical therapy, including hot pack, transcutaneous electrical nerve stimulation (TENS), and ultrasound therapy; seven of the subjects received dry needle therapy; four patients undertook a home exercise therapy program (stretching and resistance exercises); and three of the patients underwent kinesiotherapy. The results demonstrated a significant decrease in VAS and douleur neuropathique 4 (DN4) scores from the baseline and a reduction in pain levels in TENS therapy cases. Conventional TENS therapy in another study recruiting 15 patients was correlated with a significant decline in the mean pruritus score after the treatment.^22^ Additionally, Bağcıer et al.^10^ noted a significant drop in the DN4 and the 5‐D itch scale scores in an NP patient after three sessions of dry needling therapy.

In another investigation by Fonda‐Pascual et al.,^9^ utilizing a 308 nm‐excimer lamp in 11 patients with NP resulted in a significant global reduction in VAS and pruritus with no relapse after six months of follow‐up. Moreover, the procedure was safe except for inducing variable hyperpigmentation. Likewise, UVB narrow‐band application in five peers with NP was associated with substantial improvement in pruritus in all cases.^23^ Acupuncture application in 16 patients led to complete amelioration of itching in 12 and partial relief in four patients.^24^ However, six subjects experienced exacerbation after seven months and needed additional acupuncture therapy. EMS implementation in a group of four cases was associated with rapid alleviation of pain, as reported by Wang et al.^25^ However, the pain relapsed after EMS cessation for an extended period. Based on a case report by Williams et al.,^26^ surgical decompression resulted in a 50% reduction in original pain in the 1st week. The patient was also free of symptoms four months after surgical treatment.

## 4 DISCUSSION

NP is a sensory neuropathy characterized by localized pain, pruritus, paresthesia, and numbness in the back.^21^ The chronic nature of NP, consisting of remissions and flare‐ups, can negatively influence the patient's quality of life. Hence, it is imperative to correctly recognize and treat NP, particularly as it is reported to be underdiagnosed in several cases.^27^ The exact etiology of NP is still uncertain; however, it is suggested that a multifactorial process involving muscle impingement, increase in dermal innervation, and any spinal pathology may contribute to the progression of this condition by affecting the posterior cutaneous branches of T2–T6 spinal nerves.^28^ Following chronic rubbing and scratching to relieve the discomfort, hyperpigmented patches and lichenification appear on the affected part of the back. Considering dermatological or neuromuscular aspects of NP, clinicians consider various therapeutic strategies to alleviate clinical signs and symptoms with different levels of success.^27^ Therapeutic strategies for NP have been widely adapted from options utilized for other neuropathic pain conditions.^20^ Treatment options range from topical agents and systemic medications to several procedural modalities with or without pharmacological components. Nevertheless, no treatment guidelines exist for NP, and the efficacy of these options remains unclear.

Current evidence shows topical medications and physical therapy are used as first‐line treatments, followed by systemic agents in unresponsive or intolerant patients, and procedural modalities and surgical management for refractory cases of NP.^27^ Topical agents, including capsaicin, tacrolimus, and local anesthetic creams, are considered first‐line treatments to decrease pruritus. Nonetheless, topical medications are limited due to their partial effectiveness, failure of long‐term response, and adverse events that influence patient capacitance.^29^ In these cases, oral treatment options, such as gabapentin, oxcarbazepine, and amitriptyline, are prescribed. Unlike topical agents, the tolerability of oral medications was found to be acceptable. Although treatment with gabapentin successfully resulted in symptom relief,^5^ the effectiveness of drugs targeting serotonin, histamine, and inflammation was limited.^30^ Besides, maintenance dosing for long‐term use, systemic adverse reactions, possible drug interactions, and relapse after treatment cessation are among the limitations of oral pharmacological choices.^5^, ^27^, ^31^

In recalcitrant cases of NP, the application of procedural treatments led to diverse levels of response. Procedural modalities are divided into pharmacological and non‐pharmacological procedures. Pharmaco‐procedural treatments for NP include injections of lidocaine, BTX, and corticosteroid injections. On the other hand, procedural modalities without a pharmacological component were as follows: physical therapy, exercise therapy, kinesiotherapy, acupuncture and dry needling, EMS, surgical decompression, and phototherapy.

BTX‐A is a type of purified protein that cleaves synaptosomal‐associated proteins of 25 kDa (SNAP‐25) and inhibits the release of acetylcholine at the neuromuscular junction. Studies yielded different levels of efficacy regarding the results of BTX‐A injections. In detail, long‐lasting, complete resolution of symptoms was detected in several cases,^8^, ^18^ while clinical trials demonstrated no difference between BTX‐A and saline placebo.^17^ In addition to BTX‐A, intradermal injections of Rbtx‐B substantially reduced pain and paresthesia in patients with NP and other types of focal painful neuropathies.^19^ The efficacy of BTX is due to its impact on suppressing the release of glutamate, noradrenaline, and a mediator of pain and itch called substance P from afferent C fibers of dorsal root ganglion neurons.^18^ It is imperative to note that the dose of BTX shall be adjusted for each patient based on the size of the hyperpigmentation and symptomatic region.^27^ Moreover, multiple treatment sessions might be mandatory to achieve optimum relief of symptoms.^32^

Furthermore, lidocaine has been utilized for NP not only as a topical cream but also as IV infusions, local intradermal injections, and deeper injections resulting in dorsal spinal nerve block.^7^, ^20^, ^33^ Lidocaine has been largely applied in the management of both acute and chronic pain as it blocks voltage‐sensitive sodium channels, leading to suppression of peripheral and central pain transmission. Recent investigations have also shown the involvement of the immune system, calcium and potassium channels, and N‐methyl‐D‐aspartate receptors. Some studies found that the effect of lidocaine on itching was more noticeable than its pain management properties.^20^ However, further investigations are warranted to confirm these findings.

Similar to lidocaine, corticosteroids in different forms and routes of administration have been widely used for several conditions with neuropathic itch, including NP.^11^ In addition to suppressing spontaneous and ectopic discharges from injured nerves, corticosteroids exert short‐lasting inhibitory properties on transmission in normal C fibers.^34^ Previous studies reported that topical corticosteroids did not significantly affect NP, and their efficacy was limited to NP cases associated with secondary inflammation.^31^ However, procedural treatment with corticosteroids (paravertebral block with methylprednisolone acetate and bupivacaine as well as intralesional triamcinolone acetonide injections) successfully controlled NP‐associated pruritus.^6^, ^11^

Several non‐invasive procedures, such as physiotherapy, manipulative therapy, acupuncture and dry needling, analgesic electric currents, and traction, were successful in alleviating NP symptoms, including pain and itch.^10^, ^21^, ^24^ Physical therapy acts through musculoskeletal factors, leading to long‐lasting symptomatic improvement.^27^ In detail, these approaches mainly aim to reduce the compression of the dorsal branch of spinal nerves by decreasing the edema around the nerves, relaxing the muscles, and inducing the release of opioid peptides in the dorsal horn, which prohibits nociceptive information release.^24^ Physical treatments can be applied as a first‐line choice in some patients or can be combined with other options to enhance the treatment response. In cases who were unresponsive to the treatments mentioned above, surgical management can effectively resolve pain.^26^

Moreover, phototherapy and light‐based modalities led to a level of pruritus improvement. NB‐UVB is able to diminish the count of epidermal nerve fibers, which may be elevated locally in patients with NP.^23^ However, in patients with severe and chronic NP, innervation is decreased in the affected skin parts. Furthermore, the wavelength of the 308‐nm excimer lamp is similar to NB‐UVB.^9^ Another advantage of a 308‐nm excimer lamp is that it acts locally on a specific area, unlike NB‐UVB, which needs dose adjustment and imposes unnecessary irradiation on the patient.

It is important to note that NP is a rare condition; therefore, the studies assessing the treatment options have a small sample size and limited evidence. Besides, NP patients are diagnosed with diverse clinical symptoms and treated by a wide range of subspecialists. Procedural modalities have a significant role in the management of NP symptoms and can be used as first‐line, second‐line, and last‐line options. However, the effectiveness is still doubtful and needs to be studied in large‐scale clinical trials with the control group.

## 5 CONCLUSION

NP is a chronic condition with sensory abnormalities presenting with pruritus of the medial scapular borders, often accompanied by pain and post‐inflammatory hyperpigmentation.

Considering the multifactorial etiology of NP, its diagnosis and management necessitate a multidisciplinary approach. The importance of procedural modalities in NP is due to their ability to provide targeted relief, especially when topical and oral medications fail to offer adequate symptom control. Aside from topical and oral treatments, physical therapy can be considered as a first‐line option in some cases. Furthermore, pharmacological procedures and phototherapy are useful in reducing itching. It is imperative to consider that investigations on NP treatment are mostly small‐sized due to its rarity. Hence, larger‐scale clinical trials with long‐term follow‐up are required.

## CONFLICT OF INTEREST STATEMENT

The authors declared no conflict of interest.

## Supporting information

Supporting Information

Supporting Information

Supporting Information

Supporting Information

## Data Availability

All data presented in this review are available in the text and supplementary materials.
