# Supplementary material for: A systematic review of procedural modalities in the treatment of notalgia paresthetica
Source: Skin Res Technol. 2024 May 2;30(5):e13723. doi: 10.1111/srt.13723 (PMC11064992; doi:10.1111/srt.13723)
Supplement: Supplementary file 3 — Supporting Information [file SRT-30-e13723-s002.docx]

**Table S3.** The list of search strategies and final results on each database.

| **Database**  **(Search date)** | **Step** | **Search strategy** | **Number of results** |
| --- | --- | --- | --- |
| ***PubMed***  ***Nov 14^th^, 2023*** | #1 | **("notalgia paresthetica"[Title/Abstract]) OR ("notalgia*"[Title/Abstract])** | 138 |
|  | #2 | (((("Therapeutics"[Mesh]) OR ("Therapeutic*"[Title/Abstract])) OR ("Therap*"[Title/Abstract])) OR ("Treat*"[Title/Abstract])) OR ("treatment"[Title/Abstract]) | 11,418,609 |
|  | #3 | **"Review"[Publication Type] OR "Review Literature as Topic"[MeSH] OR "Systematic Review"[Publication Type] OR "Systematic Reviews as Topic"[MeSH] OR "Meta-Analysis"[Publication Type] OR "Meta-Analysis as Topic"[MeSH] OR "Network Meta-Analysis"[MeSH]** | 3,444,496 |
|  | #4 | #1 AND #2 NOT #3 | 63 |
| ***Web of Science***  ***Nov 14^th^, 2023*** | #1 | **(TS=("notalgia paresthetica")) OR TS=("notalgia*")** | 207 |
|  | #2 | (((TS=("therapy")) OR TS=("therapeutics")) OR TS=("treat*")) OR TS=("therap*") | 9,775,369 |
|  | #3 | **(((((TS=("Review")) OR TS=("Review Literature as Topic")) OR TS=("Systematic Review" )) OR TS=("Systematic Reviews as Topic" )) OR TS=("Meta-Analysis as Topic" )) OR TS=("Network Meta-Analysis")** | 2,915,706 |
|  | #4 | #1 AND #2 NOT #3 | 92 |
| ***Embase***  ***Nov 14^th^, 2023*** | #1 | ("notalgia paresthetica")/exp OR (("notalgia paraesthetica"):ti,ab,kw) OR (("notalgia paresthetica"):ti,ab,kw) | 218 |
|  | #2 | 'treat*':ti,ab,kw OR 'treatment*':ti,ab,kw OR 'therap*':ti,ab,kw OR 'therapeutics':ti,ab,kw | 11,753,100 |
|  | #3 | 'review':ti,ab,kw OR 'systematic review':ti,ab,kw OR 'meta-analysis':ti,ab,kw OR 'network meta-analysis':ti,ab,kw OR 'review':it OR 'systematic review':it OR 'meta-analysis':it OR 'network meta-analysis':it | 4,724,144 |
|  | #4 | #1 AND #2 NOT #3 | 88 |
| **Total inclusion: 243 items** | | | |
