# Supplementary material for: A systematic review of procedural modalities in the treatment of notalgia paresthetica
Source: Skin Res Technol. 2024 May 2;30(5):e13723. doi: 10.1111/srt.13723 (PMC11064992; doi:10.1111/srt.13723)
Supplement: Supplementary file 4 — Supporting Information [file SRT-30-e13723-s003.docx]

**Table S4.** The quality assessment of one clinical trial article was included in our study.

| NIH Quality Assessment Tool for Clinical Trials | | | | | | | | | | | | | | | |
| --- | --- | --- | --- | --- | --- | --- | --- | --- | --- | --- | --- | --- | --- | --- | --- |
| Study ID | Q1 | Q2 | Q3 | Q4 | Q5 | Q6 | Q7 | Q8 | Q9 | Q10 | Q11 | Q12 | Q13 | Q14 | Total Quality Score |
| Maari, 2014 | ✓ | ✓ | ✓ | ✓ | ✓ | ✓ | ✓ | ✓ | ✓ | ✓ | ✓ | ✓ | ✓ | NA | 13 (good) |

Abbreviations: NA: Not applicable

**Table S5.** The quality assessment of one before-after study with no control group articles was included in our study.

| NIH Quality Assessment Tool for Before-After (Pre-Post) Studies with No Control Group | | | | | | | | | | | | | |
| --- | --- | --- | --- | --- | --- | --- | --- | --- | --- | --- | --- | --- | --- |
| Study ID | Q1 | Q2 | Q3 | Q4 | Q5 | Q6 | Q7 | Q8 | Q9 | Q10 | Q11 | Q12 | Total Quality Score |
| Savk, 2007 | ✓ | ✓ | ✓ | ✓ | CD | ✓ | ✓ | No | ✓ | ✓ | ✓ | NA | 9 (Fair) |

Abbreviations: CD: Cannot determine; NA: Not applicable

**Table S6.** The quality assessment of four observational cohort articles was included in our study.

| NIH Quality Assessment Tool for Observational Cohort and Cross-Sectional Studies | | | | | | | | | | | | | | | |
| --- | --- | --- | --- | --- | --- | --- | --- | --- | --- | --- | --- | --- | --- | --- | --- |
| Study ID | Q1 | Q2 | Q3 | Q4 | Q5 | Q6 | Q7 | Q8 | Q9 | Q10 | Q11 | Q12 | Q13 | Q14 | Total Quality Score |
| Karasel, 2022 | ✓ | ✓ | ✓ | ✓ | No | ✓ | ✓ | NA | ✓ | No | ✓ | N | CD | ✓ | 9 (fair) |
| Fonda-Pascual, 2021 | ✓ | ✓ | ✓ | ✓ | No | ✓ | ✓ | NA | ✓ | No | ✓ | CD | ✓ | ✓ | 10 (good) |
| Mülkoǧlu, 2020 | ✓ | ✓ | ✓ | ✓ | No | ✓ | ✓ | NA | ✓ | No | ✓ | CD | ✓ | ✓ | 10 (good) |
| Stellon, 2002 | ✓ | ✓ | ✓ | ✓ | No | ✓ | ✓ | NA | ✓ | No | ✓ | CD | ✓ | ✓ | 10 (good) |

Abbreviations: CD: Cannot determine; NA: Not applicable

**Table S7.** The quality assessment of ten case reports and case series articles was included in our study.

| Methodological quality in the case series and case reports assessed by Murad et al. check | | | | | | | | | |
| --- | --- | --- | --- | --- | --- | --- | --- | --- | --- |
| **Study ID** | Q1 | Q2 | Q3 | Q4 | Q5 | Q6 | Q7 | Q8 | Total Quality Score |
| **Özcan, 2020** | ✓ | ✓ | ✓ | ✓ | No | No | ✓ | ✓ | 6 |
| **Bağcıer, 2020** | ✓ | ✓ | ✓ | ✓ | No | No | NA | ✓ | 5 |
| **Chtompel, 2017** | ✓ | ✓ | ✓ | ✓ | No | No | ✓ | ✓ | 6 |
| **Pérez-Pérez, 2014** | ✓ | ✓ | ✓ | ✓ | No | No | ✓ | ✓ | 6 |
| **Grogan, 2011** | ✓ | ✓ | ✓ | ✓ | No | No | ✓ | ✓ | 6 |
| **Williams, 2010** | ✓ | ✓ | ✓ | ✓ | No | No | NA | ✓ | 5 |
| **Wallengren, 2010** | ✓ | ✓ | ✓ | ✓ | No | No | ✓ | ✓ | 6 |
| **Pérez-Pérez, 2010** | ✓ | ✓ | ✓ | ✓ | No | No | ✓ | ✓ | 6 |
| **Wang, 2009** | ✓ | ✓ | ✓ | ✓ | No | No | NA | ✓ | 6 |
| **Weinfeld, 2007** | ✓ | ✓ | ✓ | ✓ | No | No | ✓ | ✓ | 6 |

Abbreviations: NA: Not applicable
